# A Longitudinal Study of the Association between the *LEPR* Polymorphism and Treatment Response in Patients with Bipolar Disorder

**DOI:** 10.3390/ijms23179635

**Published:** 2022-08-25

**Authors:** Hui Hua Chang, Yuan-Shuo Hsueh, Yung Wen Cheng, Huai-Hsuan Tseng

**Affiliations:** 1 Institute of Clinical Pharmacy and Pharmaceutical Sciences, College of Medicine, National Cheng Kung University, Tainan 701, Taiwan; 2 School of Pharmacy, College of Medicine, National Cheng Kung University, Tainan 701, Taiwan; 3 Department of Pharmacy, National Cheng Kung University Hospital, College of Medicine, National Cheng Kung University, Tainan 701, Taiwan; 4 Department of Pharmacy, National Cheng Kung University Hospital, Dou-Liou Branch, Yunlin 640, Taiwan; 5 Department of Medical Science Industries, College of Health Sciences, Chang Jung Christian University, Tainan 711, Taiwan; 6 Department of Psychiatry, National Cheng Kung University Hospital, College of Medicine, National Cheng Kung University, Tainan 701, Taiwan; 7 Institute of Behavioral Medicine, College of Medicine, National Cheng Kung University, Tainan 701, Taiwan

**Keywords:** bipolar disorder, *LEPR*, leptin receptor, valproate, single-nucleotide polymorphisms, pharmacogenetics

## Abstract

Patients with bipolar disorder (BD) exhibit individual variability in the treatment outcome, and genetic background could contribute to BD itself and the treatment outcome. Leptin levels significantly change in BD patients treated with valproate (VPA), but whether *LEPR* polymorphisms are associated with treatment response is still unknown. This longitudinal study aimed to investigate the associations between *LEPR* polymorphisms and VPA treatment response in BD patients who were drug naïve at their first diagnosis of BD. The single-nucleotide polymorphisms (SNPs) of *LEPR* (rs1137101, rs1137100, rs8179183, and rs12145690) were assayed, and the *LEPR* polymorphism frequencies of alleles and genotypes were not significantly different between the controls (*n* = 77) and BD patients (*n* = 130). In addition, after the 12-week course of VPA treatment in BD patients, the *LEPR* polymorphisms showed significant effects on changes in disease severity. Moreover, considering the effect of the *LEPR* haplotype, the frequency of the CAGG haplotype in BD patients was higher than that in the controls (9.3 vs. 2.9%, *p* = 0.016), and the *LEPR* CAGG haplotype was associated with a better treatment response than the other haplotypes in BD patients receiving VPA treatment. Therefore, *LEPR* polymorphisms might serve as mediators involved in the therapeutic action of VPA treatment.

## 1. Introduction

Bipolar disorder (BD) is a mental disorder that typically starts in adolescence or early adulthood [1]. The disorder is characterized by the Diagnostic and Statistical Manual of Mental Disorders (DSM) as severe bouts of recurring mania and depression. Mania is defined by a decreased need for sleep, increased hyperactivity and irritability. During depressive episodes, patients face feelings of guilt, hopelessness and suicidal thoughts. The symptom of switching between depression and mania or hypomania is a specific feature of BD [2]. The natural course of BD tends to worsen without treatment, while people with BD can lead healthy and productive lives when the illness is effectively treated. In addition, the life expectancy of individuals with BD is significantly reduced, and poor psychosocial functions, such as impaired executive functions and verbal memory function, have been found in BD [3].

Due to the individual variability in the treatment outcome of BD, more mood swings and hospitalizations result in cognitive impairment. Genetic background could contribute to mood disorders and treatment outcomes. Pharmacogenomics/pharmacogenetics is the study of the stratification of the pharmacological response to a drug by a population based on the genetic variation of that population, and applications of pharmacogenomics/pharmacogenetics include accurately predicting the response to treatment or medication-related adverse effects [4]. It can also provide some new insights into the mechanisms of drugs and the development of new therapeutic agents [5]. With construction for the entire human genome based on high-throughput single-nucleotide polymorphisms (SNPs), the detection of specific DNA sequences affecting responses to drugs can now be made possible [4,6]. In addition, C. Li reported that if a drug matches one of the following characteristics, it can be applied in pharmacogenomics/pharmacogenetics: (1) variation in drug response, (2) narrow therapeutic indices, (3) severe idiosyncratic adverse reactions, and (4) increased incidence of treatment-related complications [7]. Valproate (VPA) users in BD apparently match the first criterion. Important components of the individual variability in treatment outcome, as well as treatment outcome among BD patients treated with mood stabilizers, such as VPA, may have a basis in common genetic variation that can be revealed directly through genetic association methods [8,9,10]. Thus, we expect to determine the genetic factors implicated in mechanisms of therapeutic action and predict the treatment outcome in BD patients receiving VPA through a pharmacogenetics approach. Among mood stabilizers, VPA is usually widely used in treating BD at different stages [11] and the most prescribed drug among patients newly diagnosed with BD in Taiwan [12]. In addition, VPA demonstrates neuroprotective effects [13,14,15]. Thus, we focused on the treatment response of VPA in the current study. As a result, pharmacogenetics studies in BD patients receiving VPA are warranted.

Leptin is an essential pleiotropic hormone released by white adipose tissue, and the leptin–leptin receptor responds to changes in energy intake and regulates energy homeostasis [16,17]. Not only does leptin have a role in energy intake and expenditure, but leptin–leptin receptors also play critical roles in many other neurocognitive processes and interact with various other hormones and neurotransmitters in the CNS [18,19,20], especially in BD patients [21]. Interestingly, our previous studies, similar to other reports, have demonstrated that leptin levels were associated with BD and significantly increased after mood stabilizers treatment [22,23,24,25]. Leptin levels were significantly higher in BD patients treated with VPA than in healthy controls or in remitted BD patients. Leptin signaling depends on leptin receptor function, and variation in the leptin receptor (*LEPR*) gene directly influences receptor function. In addition, a recent study indicated an association of *LEPR* gene polymorphisms with weight gain in epilepsy patients receiving VPA [25]. However, whether *LEPR* polymorphisms correspond to treatment response in BD patients treated with VPA is still unknown. To clarify the role of *LEPR* polymorphisms in BD patients, we aimed to investigate the associations of *LEPR* polymorphisms between BD patients and controls. Furthermore, we aimed to investigate whether the *LEPR* polymorphism has an effect on treatment response in BD patients receiving VPA through this 12-week follow-up longitudinal study. We also examined whether the haplotype frequencies of *LEPR* were associated with BD itself and VPA treatment response.

## 2. Results

We recruited 130 BD patients and 77 controls, and the demographic data are shown in Table 1. There were no significant differences between BD patients and controls, except for the HAMD and YMRS scores. The mean concentration of VPA was 80.6 ± 18.0 mg/L in BD patients. In addition, the *LEPR* polymorphism frequencies of alleles and genotypes were not significantly different between the controls and BD patients (Supplementary Table S1). The genotype frequencies of *LEPR* polymorphisms in the current study did not depart significantly from the Hardy–Weinberg equilibrium in the patients with BD or in the controls. In investigating the demographic characteristics of *LEPR* polymorphisms on HAMD and YMRS scores, we found no significant differences between *LEPR* polymorphisms and disease severity in BD patients, except for *LEPR* rs1137100 (Table 2). BD patients with the AA + AG genotype of *LEPR* rs1137100 had higher YMRS scores than those with the GG genotype (10.4 ± 4.1 vs. 8.8 ± 3.9, *p* = 0.030).

**Figure 1:**
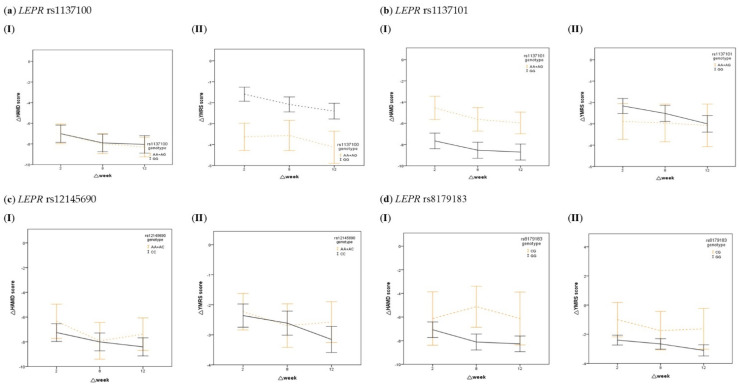
Effects of *LEPR* polymorphisms on changes in disease severity in BD patients during the 12-week VPA treatment course. Plots show the effect of *LEPR* polymorphisms (**a**) *LEPR* rs1137100, (**b**) *LEPR* rs1137101, and (**c**) *LEPR* rs12145690 and *LEPR* rs8179183 on the changes in disease severity (**I**) ∆HDRS scores and (**II**) ∆YMRS scores. (**a**) (**I**) The change in HAMD scores significantly decreased over time (*p* = 0.004), but no effects of rs1137100 on the HAMD scores were found. (**a**) (**II**) The change in YMRS scores decreased significantly over time (*p* = 0.011), and allele carriers of *LEPR* rs1137100 had a higher change in YMRS scores during the treatment course (*p* = 0.010). (**b**) (**I**) The change in HAMD scores decreased significantly over time (*p* = 0.004), but no effects of rs1137101 on the HAMD scores were found. (**b**) (**II**) The change in YMRS scores decreased significantly over time (*p* = 0.011), but no effects of rs1137101 on the YMRS scores were found. (**c**) (**I**) The change in HAMD scores decreased significantly over time (*p* = 0.003), but no effects of rs12145690 on the HAMD scores were found. (**c**) (**II**) The change in YMRS scores was not different between different genotypes during the treatment course. Patients with the CC genotype had a higher change in YMRS scores during the treatment course (*p* = 0.030). (**d**) (**I**) The change in HAMD scores decreased over time; BD patients with the GG genotype of *LEPR* rs8179183 had a higher change in HAMD scores during the treatment course (*p* = 0.037). (**d**) (**II**) The change in YMRS scores decreased significantly over time, but no effects of rs8179183 on the YMRS scores were found. Abbreviations: YMRS, 11−item Young Mania Rating Scale; HAMD, 17−item Hamilton Depression Rating Scale; BD, bipolar disorder.

After the 12-week course of VPA treatment, all of the BD patients exhibited significant reductions in the YMRS and HDRS scores (Table 1). Figure 1 shows the effects of *LEPR* polymorphisms on changes in disease severity in BD patients during the 12-week treatment course. For *LEPR* rs1137100, there were no effects on HAMD scores, while BD patients with the AA + AG genotype had a higher change in YMRS scores during the treatment course (*p* = 0.010). For *LEPR* rs1137101, there were no effects on the HAMD and YMRS scores. For *LEPR* rs12145690, there were no effects on the HAMD scores, while BD patients with the CC genotype had a higher change in YMRS scores during the treatment course (*p* = 0.030). For *LEPR* rs8179183, BD patients with the GG genotype had a higher change in HAMD scores during the treatment course (*p* = 0.037), while there were no effects on the YMRS scores.

To determine the effect of *LEPR* polymorphisms on BD and its treatment response, we further performed a haplotype analysis. Table 3 shows that the haplotype frequencies of the four polymorphisms in the *LEPR* gene between BD patients and controls and the frequency of the *LEPR* CAGG haplotype (order: rs12145690, rs1137100, rs1137101, and rs8179183) were significantly different between BD and controls (9.3 vs. 2.9%, *p* = 0.016). Furthermore, we found that the *LEPR* CAGG haplotype could not only differentiate remitters from nonremitters using HAMD scores but also responders and nonresponders using YMRS scores (Table 4 and Table 5). BD patients with the *LEPR* CAGG haplotype had more remitters (13.9% vs. 7.3%, *p* = 0.038) and responders (16.6% vs. 6.7%, *p* = 0.013) than those with other haplotypes.

## 3. Discussion

To our knowledge, this is the first longitudinal study to report associations between *LEPR* polymorphisms and VPA treatment response in BD patients who were drug naïve at their first diagnosis of BD. In the current study, the *LEPR* frequencies of alleles and genotypes were not significantly different between the controls and BD patients, and *LEPR* polymorphisms and disease severity were not different in BD patients when they were diagnosed with BD at baseline, except *LEPR* rs1137100. During the 12-week VPA treatment period, *LEPR* polymorphisms showed significant effects on changes in disease severity in BD patients. In addition, considering the effect of the *LEPR* haplotype on BD and its treatment response, we found that the frequency of the CAGG haplotype in BD patients was higher than that in the controls. Moreover, the *LEPR* CAGG haplotype was associated with a better treatment response than the other haplotypes in BD patients receiving VPA treatment. Therefore, the results of the current study suggested that the polymorphisms of *LEPR,* especially the CAGG haplotype, might serve as mediators involved in the therapeutic action of VPA treatment.

Leptin is an appetite-regulating hormone produced in white adipose tissue that acts on the hypothalamus to suppress food intake and increase energy expenditure, and it comprises 167 amino acids [17,26]. Leptin influences glucose homeostasis by direct action on the liver and muscle and pancreas β cells [27]. Leptin decreases glucose and insulin levels by enhancing peripheral glucose uptake and insulin sensitivity [28]. In addition, studies in humans have provided evidence about the role of leptin in mood disorders [29,30]. Lower plasma leptin levels were observed in patients with bipolar disorder [31]. However, our previous study found that bipolar patients who received valproate had increased peripheral leptin levels [22], and VPA has been confirmed to stimulate appetite and decrease thermogenesis in the hypothalamus, resulting in weight gain and metabolic syndrome [32]. Overall, leptin was found to be another common mediator regulating mood disorder and metabolic homeostasis [29]. In our study, although we did not investigate whether leptin levels were associated with BD itself or with metabolic indices in BD patients, we found that *LEPR* polymorphisms influence the individual treatment response. The relevant mechanisms of leptin and *LEPR* in BD need to be clarified.

Leptin exerts its function by binding to leptin receptors [17]. The leptin receptor is a member of the cytokine receptor superfamily, it is a single transmembrane protein, and its different isoforms are distributed in different tissues. For example, Ob-Rb, which is a biologically active isoform of the leptin receptor, is primarily expressed in the hypothalamus and is also found in tissues and cells that regulate glucose homeostasis, such as pancreatic β cells [33]. Leptin receptors are encoded by the *LEPR* gene [27]. *LEPR* is located on chromosome 1p31, and it includes more than 70 thousand base pairs and consists of 20 exons. According to previous studies, several polymorphisms of the *LEPR* gene, such as Arg109Lys (rs1137100), Gln223Arg (rs1137101) and Asn656Lys (rs8179183), were reported to be associated with type 2 diabetes [34]. On the other hand, rs1137101 was associated with weight gain in epilepsy patients receiving VPA [25]. Furthermore, a study reported that the polymorphism of the *LEPR* gene was associated with resistance to antidepressant treatment in MDD patients [35]. Therefore, we selected four *LEPR* SNPs (rs1137100, rs1137101, rs8179183, and rs12145690) and investigated the associations between the polymorphisms and treatment outcome in BD patients during the 12-week valproate treatment course. Our results indicated that polymorphisms of the *LEPR* gene were associated with treatment response in patients receiving VPA, and we would like to further investigate the possible therapeutic mechanism of the *LEPR* gene as leptin and leptin receptors might be involved in the pathophysiology of BD. Moreover, histone deacetylase (HDAC) is known to be a direct target of VPA, and recent studies have suggested that the modulation of G-protein signaling by VPA might occur through its effects on HDAC and gene transcription [36,37,38]. HDAC inhibitors have been noted to play roles in glucose homeostasis and metabolic regulation [39,40]. Therefore, it is important to know whether the therapeutic mechanism of VPA is correlated with the effect on leptin regulation through HDAC in BD.

In addition to various individual differences in treatment response in BD, previous studies have shown that patients with BD are prone to metabolic syndrome, which may increase the risk of cardiovascular disease and diabetes mellitus. The comorbidity of BD and metabolic syndrome has been associated with a worse disease course [41]. Previously, a review summarized the prevalence of MS in BD patients from different countries, and the prevalence rate ranged from 16% to 60% [22,42]. Therefore, investigating the causal relationship between BD and metabolic syndrome is an important issue. Previous studies have noted that some factors may increase the risk of bipolar patients developing metabolic syndrome. These factors include increased proinflammatory cytokine production [43] and psychiatric medications, such as mood stabilizers, valproate (VPA), and antipsychotics [23,44]. Genetic effects [45,46], such as *GNB3*, and environmental factors, such as smoking and education level, may contribute to the risk for metabolic syndrome in BD. Another common mediator of energy balance and mood symptoms might be leptin, as leptin and leptin receptors play roles in obesity [26]. Here, we demonstrated that polymorphisms of *LEPR* play roles in changes in disease severity during treatment response. Furthermore, it is interesting to investigate whether genetic biomarkers could predict treatment-related metabolic syndrome in BD patients and provide intervention early. Whether *LEPR* polymorphisms are also associated with metabolic syndrome in BD patients receiving VPA remains to be further investigated.

Although the data were presented carefully, this study has certain limitations. The first limitation is the relatively small sample size of BD patients and controls from a single site. Although there was a small sample size, the frequencies of the *LEPR* polymorphisms in controls were consistent with those presented in the Ensembl project for participants of Asian ethnicity (Supplementary Table S1). The second limitation was that the study period was short, 12 weeks of observation after the initiation of VPA treatment. The third limitation was the lack of controlling for some confounding factors, such as diet, smoking, drinking, exercise, socioeconomic status and general health status, which might influence treatment response in BD. The fourth limitation was that all BD patients were drug naïve and at their first diagnosis of BD, criteria aimed at decreasing the bias and confounding factors. Thus, the current results might not be extrapolated to other BD patients. Further study is needed to clarify whether *LEPR* polymorphisms could be a useful clinical indicator for monitoring treatment response and even metabolic disturbance in BD patients.

In conclusion, the present study showed that BD patients exhibited significant reductions in the YMRS and HDRS scores, while *LEPR* polymorphisms showed significant effects on changes in disease severity in BD patients during the VPA treatment course. Regarding the *LEPR* haplotype, the CAGG haplotype could be associated with BD itself and with VPA treatment response. This finding might help to develop tailored individual therapies for BD patients to optimize VPA treatment, as BD is a severe chronic psychiatric disease that requires maintenance therapy with mood stabilizers. Previously, most pharmacogenetic studies of BD have focused on the response to lithium, and few studies have focused on VPA, especially in BD patients who are drug naïve [47,48]. Therefore, our study demonstrated that the pharmacogenetics approach can enable clinical utility. Our long-term goal is to establish personalized treatment programs for patients with BD by leveraging a pharmacogenetics approach.

## 4. Materials and Methods

### 4.1. Subjects

The Institutional Review Board for the Protection of Human Subjects at National Cheng Kung University Hospital (NCKUH) approved the research protocol (IRB No. A-ER-104-031). All participants (18 to 65 years old) recruited consecutively (from March 2015 to August 2019) from outpatient settings at the National Cheng Kung University Hospital and provided written informed consent regarding their willingness to participate in the research. In addition, to avoid possible confounding factors, we also recruited controls from the community after individuals with mental illnesses were excluded by a senior psychiatrist using the Chinese version of the Mini International Neuropsychiatry Interview. All of the BD patients were initially evaluated in an interview by an attending psychiatrist using the Chinese Version of the Modified Schedule of Affective Disorder and Schizophrenia–Life Time, which has good interrater reliability, to determine the Diagnostic and Statistical Manual of Mental Disorders, Fifth Edition diagnoses (DSM-5). The same rater conducted the initial and subsequent ratings for each patient and was blinded to the subject’s genotype. The patients also met the following inclusion criteria: (i) diagnosis of BD II using the 2-day minimum for hypomania [49,50], (ii) major depressive status at the time of study entry, with a 17-item Hamilton Depression Rating Scale (HDRS) score > 15, and (iii) drug-naïve with a diagnosis of BD for the first time, with no history of mood stabilizer treatment. The exclusion criteria included (i) the presence of other major psychiatric illnesses (such as schizophrenia, BD I), (ii) a history of substance or alcohol abuse or dependence, (iii) severe physical illness, (iv) previous or ongoing treatment for metabolic disturbance, and (v) a history of psychotropic agent use. To minimize the effect of ethnic bias, we recruited only Taiwanese participants who were unrelated.

After enrollment in this study, the patients received VPA 500 to 1500 mg orally per day for 12 weeks. Serum trough concentration of VPA was assessed by the homogeneous enzyme immunoassay method at the laboratory of the Pathology Research Center of NCKUH [51]. Concomitant fluoxetine (20 mg/day) was permitted to treat depressive symptoms, and lorazepam (<8 mg) was used for night-time sedation and to treat agitation and insomnia during the study, the dosage of which was adjusted according to the clinical manifestation and the patient’s tolerance. To avoid valproate use in pregnant women, subjects meeting the following criteria are not prescribed to receive valproate treatment according to the guidance Taiwan Food and Drug Administration: (i) during pregnancy for the treatment of bipolar disorder, and (ii) women of childbearing potential unless they have birth control (contraception). The mood of each patient was evaluated using the HDRS and the 11-item Young Mania Rating Scale (YMRS) at baseline and 12 weeks after the initiation of treatment. Predefined cutoffs for remission were a HAMD or YMRS score ≤ 7 [52,53], and those for response were the percentage change in HAMD score (defined as ∆HAMD score %) or percentage change in YMRS score (defined as ∆YMRS score %) ≤ −50%.

$$△\mathrm{HAMD}\;\mathrm{score}\%\;=\frac{\left(HAMD\;score\;at\;12\;weeks\right)-\left(HAMD\;score\;at\;baseline\right)}{HAMD\;score\;at\;baseline}\;△\mathrm{YMRS}\;\mathrm{score}\%\;=\frac{\left(YMRS\;score\;at\;12\;weeks\right)-\left(YMRS\;score\;at\;baseline\right)}{YMRS\;score\;at\;baseline}$$

### 4.2. Genotyping

Ten milliliters of whole blood were withdrawn from the antecubital vein of each participant using a heparin tube. A buffy coat was isolated from plasma and red blood cells after centrifugation at 3000× *g* for 15 min at 4 °C and immediately stored at −80 °C. Genomic DNA was extracted from each buffy coat sample using a QIAamp DNA blood kit (Qiagen, Hilden, Germany) according to the manufacturer’s instructions. The quality of the extracted genomic DNA was checked by agarose gel electrophoresis analysis. The DNA was stored at −80 °C until use. The SNPs of the *LEPR* gene (rs1137101, rs1137100, rs8179183, and rs12145690) were chosen based on literature reports [25,54,55,56] and were analyzed using commercially available TaqMan SNP Genotyping Assays (Applied Biosystems, Foster City, CA, USA) according to the manufacturer’s instructions. Amplification and dissociation were conducted using an ABI 7900HT Fast Real-Time PCR System (Applied Biosystems, Foster City, CA, USA). The PCR system automatically calculated the negative derivative of the change in fluorescence. The SNP genotype of each tested sample was determined by a software program and confirmed manually. In case of disagreement, the analysis was repeated.

### 4.3. Statistical Analysis

Statistical analysis was performed using the Statistical Package for Social Sciences 19.0 (SPSS Inc., Chicago, IL, USA). Categorical variables are expressed as numbers and percentages, and continuous variables are expressed as the means ± standard deviations (SDs) unless otherwise specified. Categorical variables were assessed using chi-square tests, and continuous variables were assessed using t tests. In addition, intent-to-treat analyses using the mixed-effects model for repeated measures were used to analyze the baseline disease severity as the primary outcome at baseline and after VPA treatment. Data were analyzed using the last observation carried forward (LOCF) method, in which the last observation was entered for missing visits [57]. We further investigated the effects of the polymorphisms of *LEPR* on the changes in disease severity in BD patients. The observed and expected genotype frequencies were compared using a chi-square goodness-of-fit test to ensure that the loci did not depart from the Hardy–Weinberg equilibrium. Genotype and allele frequencies in different groups were compared through chi-square tests. Dominant models were used to compare the differences in disease severity, which were calculated by using Student’s t test. In addition, we investigated whether the *LEPR* polymorphism had an effect on treatment outcomes during the 12-week VPA treatment course. We used repeated-measures ANOVA with the four visit values of HAMD and YMRS as dependent variables, while the intervention and the *LEPR* polymorphism × intervention interaction were independent variables. Haplotype frequencies and linkage disequilibrium coefficients (D’) were assessed by the expectation-maximization (EM) algorithm using SNP chi-square tests. The odds ratio and its 95% confidence interval between groups were calculated. The level of significance was set at 0.05 for two-sided tests.

## Figures and Tables

**Table 1:** Demographic characteristics and measurements between controls and BD patients.

	Controls (*n* = 77)	BD Patients (*n* = 130)	BD Patients after Treatment	95% CI ^a^	t ^a^/χ^2^	*p* Value ^a^	95% CI ^b^	t ^b^	*p* Value ^b^
Age (y) (Range, min–max)	31.0 ± 10.7 (19–59)	32.1 ± 11.6 (18–64)	–	−2–4.2	−0.690	0.491	–	–	–
Gender, female (%)	43 (55.8)	66 (50.8)	–	−0.1–0.2	0.564	0.453	–	–	–
HAMD scores	3.2 ± 1.6	19.4 ± 5.5	11.4 ± 6.9	14.9–17.4	−25.327	<0.001 *	6.8–9.2	12.911	<0.001 *
YMRS scores	4.1 ± 0.4	9.2 ± 4.0	6.4 ± 3.4	4.3–6	−11.428	<0.001 *	2.2–3.6	8.147	<0.001 *

Abbreviations: YMRS, 11-item Young Mania Rating Scale; HAMD, 17-item Hamilton Depression Rating Scale. ^a^ Compared with healthy controls. ^b^ Paired t test in BD patients at baseline and after VPA treatment. * *p* < 0.05.

**Table 2:** The YMRS and HDRS scores in controls and BD patients subgrouped by *LEPR* polymorphisms.

	Controls					BD				
rs1137100	AG (*n* = 26)	GG (*n* = 51)	t	95% CI	*p*	AA + AG (*n* = 46)	GG (*n* = 84)	t	95% CI	*p*
HAMD scores	3.4 ± 1.6	3.2 ± 1.6	0.498	−0.6–0.9	0.620	19.3 ± 6.5	19.4 ± 5.2	−0.141	−2.2–1.9	0.888
YMRS scores	4.2 ± 0.5	4.1 ± 0.3	0.815	−0.1–0.3	0.418	10.4 ± 4.1	8.8 ± 3.9	2.188	0.2–3.0	0.030 *
rs1137101	AA + AG (*n* = 20)	GG (*n* = 57)				AA + AG (*n* = 27)	GG (*n* = 103)			
HAMD scores	3.6 ± 1.7	3.1 ± 1.5	1.261	−0.3–1.3	0.211	18.8 ± 6.7	19.5 ± 5.4	−0.580	−3.1–1.7	0.563
YMRS scores	4.1 ± 0.4	4.1 ± 0.4	−0.052	−0.2–0.2	0.958	9.7 ± 4.6	9.3 ± 3.8	0.508	−1.3–2.2	0.612
rs12145690	AA + AC (*n* = 25)	CC (*n* = 52)				AA + AC (*n* = 26)	CC (*n* = 104)			
HAMD scores	3.3 ± 1.6	3.2 ± 1.6	0.382	−0.6–0.9	0.703	18.7 ± 5.5	19.5 ± 5.7	−0.630	−3.2–1.7	0.530
YMRS scores	4.2 ± 0.5	4.1 ± 0.3	0.889	−0.1–0.3	0.377	9.7 ± 4	9.3 ± 4.0	0.476	−1.3–2.2	0.635
rs8179183	CG (*n* = 9)	GG (*n* = 68)				CG (*n* = 8)	GG (*n* = 122)			
HAMD scores	3.8 ± 1.8	3.2 ± 1.5	1.135	−0.5–1.7	0.260	19.6 ± 4.9	19.4 ± 5.7	0.128	−3.8–4.4	0.898
YMRS scores	4.4 ± 0.7	4.1 ± 0.3	1.576	−0.2–0.9	0.152	8.9 ± 3.4	9.4 ± 4	−0.377	−3.4–2.3	0.707

Abbreviations: YMRS, 11-item Young Mania Rating Scale; HAMD, 17-item Hamilton Depression Rating Scale. * *p* < 0.05.

**Table 3:** Haplotype frequencies of the 4 polymorphisms in the *LEPR* gene in BD patients and healthy controls.

Haplotype ^#^		BD Patients	Healthy Controls	X^2^	*p*	Odds Ratio	95% CI
1	CGGG	69.9	68.1	>0.001	0.995	1.0	0.6–1.6
2	AGGG	7.4	11.2	1.964	0.161	0.6	0.3–1.2
3	CAGG	9.3	2.9	5.756	0.016 *	3.3	1.2–9.2
4	CAAG	6.1	7.6	0.429	0.512	0.8	0.4–1.7
5	CGAG	2.5	1.5	0.378	0.539	1.6	0.4–7.2
6	AAAC	1.4	1.8	0.129	0.720	0.8	0.2–3.6
7	AAGG	0.9	1.8	0.688	0.407	0.5	0.1–2.7
8	CGGC	1.3	1.2	>0.001	0.985	1.0	0.2–6.3

The SNP order in each haplotype. 1: rs12145690 (A/C); 2: rs1137100 (A/G); 3: rs1137101 (A/G); 4: rs8179183 (C/G). The comparison of frequencies between BD patients and controls was performed using a chi-square test. ^#^ Frequency threshold was set to >1%. * *p* < 0.05.

**Table 4:** Haplotype frequencies of the 4 polymorphisms in the *LEPR* gene in HAMD remitters and nonremitters.

Haplotype ^#^		HAMD Remitter (%)	HAMD Nonremitter (%)	X^2^	*p*	Odds Ratio	95% CI
1	CGGG	69.6	69.4	0.009	0.923	1.0	0.6–1.7
2	CAGG	13.9	7.3	2.912	0.038 *	2.0	1.9–4.6
3	AGGG	7.1	8.1	0.103	0.748	0.9	0.3–2.2
4	CAAG	5.2	6.3	0.163	0.687	0.8	0.3–2.4
5	CGAG	2.2	2.8	0.093	0.761	0.8	0.2–3.9
6	AAAC	0	2.5	2.536	0.111	0	–
7	CGGC	1	1.5	0.130	0.718	0.7	0.1–6.8

The SNP order in each haplotype. 1: rs12145690 (A/C); 2: rs1137100 (A/G); 3: rs1137101 (A/G); 4: rs8179183 (C/G). The comparison of frequencies between remitters and nonremitters was performed using a chi-square test. ^#^ Frequency threshold: >1%. * *p* < 0.05.

**Table 5:** Haplotype frequencies of the 4 polymorphisms in the *LEPR* gene in YMRS responders and nonresponders.

Haplotype ^#^		YMRS Responder (%)	YMRS Nonresponder (%)	X^2^	*p*	Odds Ratio	95% CI
1	CGGG	63.3	72.3	2.283	0.131	0.6	0.4–1.1
2	CAGG	16.6	6.7	6.108	0.013 *	2.8	1.2–6.3
3	AGGG	7.1	8.0	0.074	0.786	0.9	0.3–2.4
4	CAAG	6.4	5.7	0.044	0.834	1.1	0.4–3.4
5	CGAG	2.7	2.5	0.008	0.929	1.1	0.2–5.5
6	AAAC	1.2	1.7	0.085	0.771	0.7	0.1–7.1
7	CGGC	1.2	1.3	0.006	0.937	0.9	0.1–9.5

The SNP order in each haplotype. 1: rs12145690 (A/C); 2: rs1137100 (A/G); 3: rs1137101 (A/G); 4: rs8179183 (C/G). The comparison of frequencies between responders and nonresponders was performed using a chi-square test. ^#^ Frequency threshold was set to >1%. * *p* < 0.05.

## Data Availability

The data that support the findings of this study are available from National Cheng Kung University. Restrictions apply to the availability of these data, which were used under license for this study. The data presented in this study are available on request from the corresponding author with the permission of National Cheng Kung University.

## Supplementary Materials

The following supporting information can be downloaded at: https://www.mdpi.com/article/10.3390/ijms23179635/s1.
