# Supplementary material for: A Longitudinal Study of the Association between the LEPR Polymorphism and Treatment Response in Patients with Bipolar Disorder
Source: Int J Mol Sci. 2022 Aug 25;23(17):9635. doi: 10.3390/ijms23179635 (PMC9455965; doi:10.3390/ijms23179635)
Supplement: Supplementary file 1 [file ijms-23-09635-s001.zip › ijms-1864785-supplementary.pdf]

International Journal of *Molecular Sciences*

MDPI

Supplementary Table S1. The allele frequencies of the *LEPR* polymorphism in controls and BD patients.

LEPR		Healthy controls (n= 77)				BD II patients (n= 130)					HapMap-JPT	
SNP rs#	Allele (major/minor)	HO	HE	WT	HWE	HO	HE	WT	HWE	p^3	MAF	p^4
		N (%)	N (%)	N (%)	p^1	N (%)	N (%)	N (%)	p^2			
rs1137100	G/A	0 (0.0)	26 (33.8)	51 (66.2)	0.075	2 (1.5)	44 (33.8)	84 (64.7)	0.157	0.548	0.244	0.147
rs1137101	G/A	1 (1.3)	19 (24.7)	57 (74.0)	0.676	1 (0.8)	26 (20.0)	103 (79.2)	0.643	0.672	0.165	0.360
rs12145690	C/A	1 (1.3)	24 (31.2)	52 (67.5)	0.332	2 (1.5)	24 (18.5)	104 (80.0)	0.662	0.119	0.188	0.575
rs8179183	G/C	0 (0.0)	9 (11.7)	68 (88.3)	0.586	0 (0.0)	8 (6.2)	122 (93.8)	0.717	0.128	0.128	0.108

Allele order: major/minor. [1] HWE of healthy controls. [2] HWE of BD patients. [3] Comparison of the genotypic distribution of BD patients with healthy controls using the χ^2 goodness-of-fit test. [4] Comparison of the allele frequency in healthy controls with HapMap-JPT using the χ^2 goodness-of-fit test. Abbreviations: HO, homozygous; HE, heterozygous; WT, wild-type; HWE, Hardy–Weinberg equilibrium; MAF, minor allele frequency; JPT, Japanese in Tokyo.
